# Supplementary material for: Chromothripsis during telomere crisis is independent of NHEJ, and consistent with a replicative origin
Source: Genome Res. 2019 May;29(5):737–49. doi: 10.1101/gr.240705.118 (PMC6499312; doi:10.1101/gr.240705.118)
Supplement: Supplemental Material [file supp_gr.240705.118_Supplemental_file_1.zip › contigs/annotated_contigs/DB50/contig.2.DB50_length_675_mean_cov_7.45925925926.docx]

**DB50_length_675_mean_cov_7.45925925926**

```
TGGGAGGCCAAGGCAGGCAGATCACCTGAGGGTCAGGAGTTCAAGACCAGCCTGGCCAACATGGCAAAACCCCATCTCTACTAAAAATA
 >chr15:76668080-76668437 + E=5e-203
CAAAAAATTAGCCAGATGTGGTGGCGGTTGCCTGTAATCCCAGCTACTTGGGAGGCTGAGGCATGAGAATCGCTTGAACCTGGGAGGCA

GAGGTTGCAGTGAGCCGAGATTGCACCACTGCATTCCAGCCTGGGTGATAGAGCGAGCCTCTGTCTCCAAAAGAAAAAAAATCTATTTG

AAGAGAAGGAGCTAGAGGCATATTCCTGCAGTCTGGTTAAAGGGCTTTACAGAGGACATTTGGGTTCCAGCCCGCTAGCCTGCCTTTTC

T|GC|CAATCATATTTAATCCTCAAATAGTCTTGTGAGGTAGGTGTTCTTAATCTCTATTTTACAGATGAGGAAATGGAGTCTTAAGTG
    >chr15:76670313-76670631 + E=1e-167
TTCAAATGACTTGTCTAAGGTCATACAATTTGAAGAGGTAATGTGATGGCTCAAAGTCAGGTCATTCTGACTCCAGCTTCAGGGGTCTA

ATGAGGATGCCTCCCTGTAACAGCTCTGTATTCTTGTGATGCATTCGAACCCAAATCATCAACATGTCTTATATCATCTTGTCTAACTC

TGCAGCCGGTCAGACAATTTATCTGTTTTTTTCTTCAATTCCTTTGAGCCTCCC
```
